# Supplementary material for: Hearing Function: Identification of New Candidate Genes Further Explaining the Complexity of This Sensory Ability
Source: Genes (Basel). 2021 Aug 10;12(8):1228. doi: 10.3390/genes12081228 (PMC8394865; doi:10.3390/genes12081228)
Supplement: Supplementary file 1 [file genes-12-01228-s001.zip › supplementary_files/Supplementary_Cohorts_Description.pdf]

Description of the cohorts

Populations from the Italian Network of Genetic Isolates (INGI) project

The INGI project is a collaboration between research institutions in Italy aimed at reconstructing the molecular bases of complex traits by investigating genetically isolated Italian populations. Studies were conducted referring to a common operational protocol. In each population, genotype samples were collected, alongside a detailed anamnesis, more than 120 biochemical parameters and 400 phenotypes, including anthropometric measures, lifestyle habits, diseases and pure-tone audiometry [1]. Data from three of these populations were available for this work: INGI Friuli-Venezia Giulia, INGI Val Borbera and INGI Carlantino.

The INGI Friuli-Venezia Giulia cohort (FVG) is a collection of samples coming from six small villages (Clauzetto, Erto, Illegio, Resia, San Martino del Carso and Sauris) located in north-eastern Italy, in the Friuli-Venezia Giulia region [2].

The INGI Val Borbera cohort (VBI) consists of samples collected from seven small villages in the Borbera Valley, a geographically isolated valley located within the Apennine Mountains, in the southern part of Piedmont, in north-western Italy. The valley is inhabited by approximately 3,000 descendants from the original population [3].

The INGI Carlantino cohort (CAR) consists of samples collected in a small village of the same name, situated in the northern part of Puglia region, in southern Italy [2].

Silk Road (SR)

Silk Road cohort is composed of samples collected in 2010 by the Marco Polo Scientific Expedition (promoted by the University of Trieste) from small rural communities located in Caucasus and Central Asia, along the historical trade route known as Silk Road. Each participant underwent a careful clinical (psychological, neurological, cardiological, …) examination and an audiometric test; data on lifestyle and food habits was also collected [4].

Age-Related Hearing Impairment Study Antwerp (AWP)

A population-based sample was obtained through population registries made available by the local city councils. To make the population ethnically homogenous, we requested that at least three out of the four grandparents originated from the same region as the study subject. All responding subjects underwent clinical examination, otoscopy and completed a detailed questionnaire on medical history and exposure to environmental risk factors. A list of all questions and answers used in this study is available on request.

Strict exclusion criteria were applied to exclude persons having or having had a condition that possibly leads to hearing impairment. No phenotypic inclusion criteria were used for the sample collection. Subjects with ear diseases, possible monogenic forms of hearing impairment or other major pathologies with a possible influence on hearing, were excluded. The main goal was to study hearing impairment in healthy subjects and therefore persons with multiple hospitalizations were excluded. The complete list of exclusion criteria was previously reported [5].

Rotterdam Study (RS)

The Rotterdam Study is a prospective, population-based cohort study among subjects living mainly in the Ommoord district in the city of Rotterdam, The Netherlands. Started in 1990, the study is aimed at the investigation of cardiovascular, endocrine, hepatic, neurological, ophthalmic, psychiatric, dermatological, otolaryngological, locomotor, and respiratory diseases. During the years it has had several recruitment extensions (referred to as RS 1, 2, 3, …), expanding at the same time the range of collected phenotypes [6]. In 2011 hearing assessment by means of pure-tone audiometry was implemented in the study protocol [7].

Framingham Heart Study (FHS)

The Framingham Heart Study is a prospective longitudinal investigation of the development of atherosclerosis and its clinical sequelae [8]. Study participants were recruited at three time periods. The study was initiated in 1948-50 with the recruitment of 5,209 individuals ages 28-62 (including some spouse pairs, parent-offspring pairs and siblings) for the purpose of investigating the multiple factors involved in the development of cardiovascular disease. This group, known as the Original Cohort, has been examined every two years with a total of thirty-two examinations to date. In 1971-1975, offspring of the Original Cohort and the offspring spouses were recruited to examine among other goals the familial components of cardiovascular disease and its risk factors. In 2002-2005, the third generation (children of the Offspring and grandchildren of the Original Cohort) was recruited. The Offspring Cohort totaled 5,124 and the Third Generation totaled 4,095 at recruitment and have been examined every 4 to 8 years. The Offspring Cohort now has 9 examinations completed and the Third Generation has 2 examinations completed.

Between 1973 and 1975, hearing examinations were conducted on 2,293 members of the original cohort, and between 1995 and 1999, identical examinations were conducted on 2,262 members of the offspring cohort. Standard pure-tone audiograms were obtained on all participants using environments and meeting American National Standards Institute standards.

Salus in Apulia Study (SIA)

The “Salus in Apulia Study” is an ongoing population-based prospective cohort comprising 2,472 individuals aged 65 years old or older and residents in Castellana Grotte, a town located near Bari, Puglia, in the Southeast of Italy. It focused on the sequence of lifestyle including diet, frailty, and other age-related impairments and age-related disease outcomes. In detail, Salus is a public health initiative funded by the Italian Ministry of Health and Apulia Regional Government and carried on at IRCCS “S. De Bellis” that combines data from two previous populations: the baseline data (MICOL3, M3) were recorded from 2003 to 2005 and the follow-up data from 2013 to 2015 (GreatAGE Study - MICOL4, M4). The GreatAGE-M4 study has been described elsewhere [9]. The invitation included also subjects of the MICOL studies that were in the respective age range above 64 years. In the GreatAge-M4 examination, in addition to the assessment of clinical and lifestyle aspects, sensory-related outcomes have been also evaluated together with neuropsychological features and genetic components. The study adhered to the “Standards for Reporting Diagnostic Accuracy Studies” (STARD) guidelines (http://www.stard-statement.org/), the “Strengthening the Reporting of Observational Studies in Epidemiology” (STROBE) guidelines (https://www.strobe-statement.org/).

REFERENCES

(1) Girotto, G.; Pirastu, N.; Sorice, R.; Biino, G.; Campbell, H.; d’Adamo, A. P.; Hastie, N. D.; Nutile, T.; Polasek, O.; Portas, L.; Rudan, I.; Ulivi, S.; Zemunik, T.; Wright, A. F.; Ciullo, M.; Hayward, C.; Pirastu, M.; Gasparini, P. Hearing Function and Thresholds: A Genome-Wide Association Study in European Isolated Populations Identifies New Loci and Pathways. *Journal of Medical Genetics* **2011**, *48* (6), 369–374. https://doi.org/10.1136/jmg.2010.088310.

(2) Esko, T.; Mezzavilla, M.; Nelis, M.; Borel, C.; Debniak, T.; Jakkula, E.; Julia, A.; Karachanak, S.; Khrunin, A.; Kisfali, P.; Krulisova, V.; Kučinskiené, Z. A.; Rehnström, K.; Traglia, M.; Nikitina-Zake, L.; Zimprich, F.; Antonarakis, S. E.; Estivill, X.; Glavač, D.; Gut, I.; Klovins, J.; Krawczak, M.; Kučinskas, V.; Lathrop, M.; Macek, M.; Marsal, S.; Meitinger, T.; Melegh, B.; Limborska, S.; Lubinski, J.; Paolotie, A.; Schreiber, S.; Toncheva, D.; Toniolo, D.; Wichmann, H. E.; Zimprich, A.; Metspalu, M.; Gasparini, P.; Metspalu, A.; D’Adamo, P. Genetic Characterization of Northeastern Italian Population Isolates in the Context of Broader

European Genetic Diversity. *European Journal of Human Genetics* **2013**, *21* (6), 659–665. https://doi.org/10.1038/ejhg.2012.229.
(3) Cocca, M.; Barbieri, C.; Concas, M. P.; Robino, A.; Brumat, M.; Gandin, I.; Trudu, M.; Sala, C. F.; Vuckovic, D.; Girotto, G.; Matullo, G.; Polasek, O.; Kolčić, I.; Gasparini, P.; Soranzo, N.; Toniolo, D.; Mezzavilla, M. A Bird's-Eye View of Italian Genomic Variation through Whole-Genome Sequencing. *Eur J Hum Genet* **2020**, *28* (4), 435–444. https://doi.org/10.1038/s41431-019-0551-x.
(4) Mezzavilla, M.; Vozzi, D.; Pirastu, N.; Girotto, G.; d'Adamo, P.; Gasparini, P.; Colonna, V. Genetic Landscape of Populations along the Silk Road: Admixture and Migration Patterns. *BMC Genetics* **2014**, *15* (1), 1–10. https://doi.org/10.1186/s12863-014-0131-6.
(5) Van Eyken, E.; Van Laer, L.; Fransen, E.; Topsakal, V.; Lemkens, N.; Laureys, W.; Nelissen, N.; Vandevelde, A.; Wienker, T.; Van De Heyning, P.; Van Camp, G. KCNQ4: A Gene for Age-Related Hearing Impairment? *Human Mutation* **2006**, *27* (10), 1007–1016. https://doi.org/10.1002/humu.20375.
(6) Ikram, M. A.; Brusselle, G. G. O.; Murad, S. D.; van Duijn, C. M.; Franco, O. H.; Goedegebure, A.; Klaver, C. C. W.; Nijsten, T. E. C.; Peeters, R. P.; Stricker, B. H.; Tiemeier, H.; Uitterlinden, A. G.; Vernooij, M. W.; Hofman, A. The Rotterdam Study: 2018 Update on Objectives, Design and Main Results. *European Journal of Epidemiology* **2017**, *32* (9), 807–850. https://doi.org/10.1007/s10654-017-0321-4.
(7) Rigters, S. C.; Van Der Schroeff, M. P.; Papageorgiou, G.; Baatenburg De Jong, R. J.; Goedegebure, A. Progression of Hearing Loss in the Aging Population: Repeated Auditory Measurements in the Rotterdam Study. *Audiology and Neurotology* **2019**, *23* (5), 290–297. https://doi.org/10.1159/000492203.
(8) Tsao, C. W.; Vasan, R. S. Cohort Profile: The Framingham Heart Study (FHS): Overview of Milestones in Cardiovascular Epidemiology. *Int. J. Epidemiol.* **2015**, *44* (6), 1800–1813. https://doi.org/10.1093/ije/dyv337.
(9) Sardone, R.; Battista, P.; Donghia, R.; Lozupone, M.; Tortelli, R.; Guerra, V.; Grasso, A.; Griseta, C.; Castellana, F.; Zupo, R.; Lampignano, L.; Sborgia, G.; Capozzo, R.; Bortone, I.; Stallone, R.; Fiorella, M. L.; Passantino, A.; Giannelli, G.; Seripa, D.; Panza, F.; Logroscino, G.; Quaranta, N. Age-Related Central Auditory Processing Disorder, MCI, and Dementia in an Older Population of Southern Italy. *Otolaryngol Head Neck Surg* **2020**, *163* (2), 348–355. https://doi.org/10.1177/0194599820913635.
