# Supplementary figures and images for: Hearing Function: Identification of New Candidate Genes Further Explaining the Complexity of This Sensory Ability
Source: Genes (Basel). 2021 Aug 10;12(8):1228. doi: 10.3390/genes12081228 (PMC8394865; doi:10.3390/genes12081228)

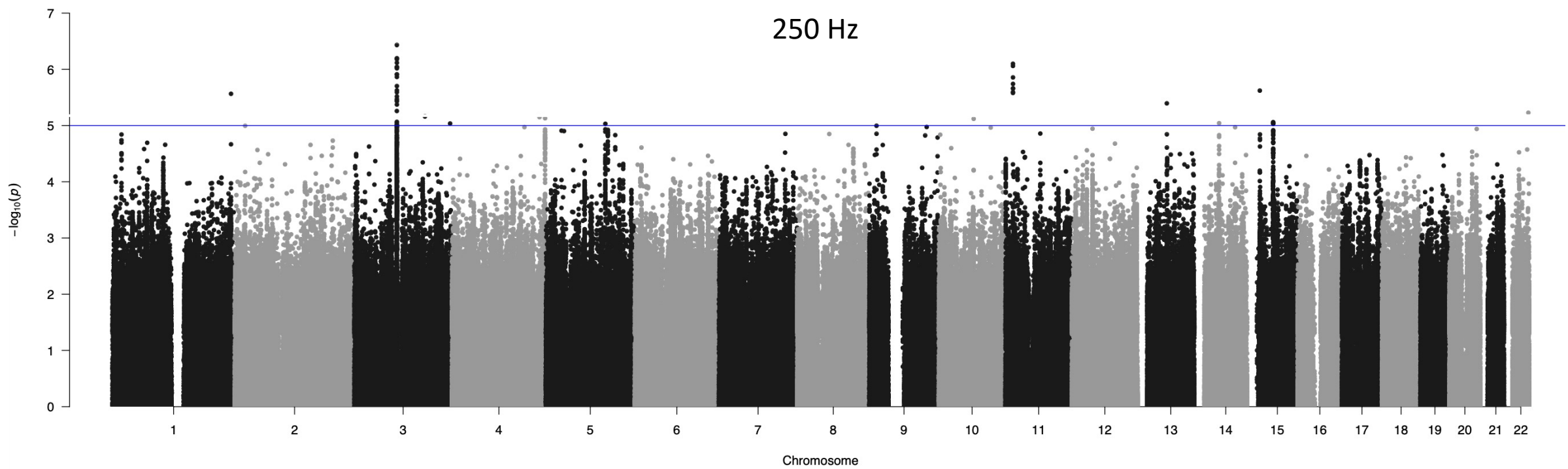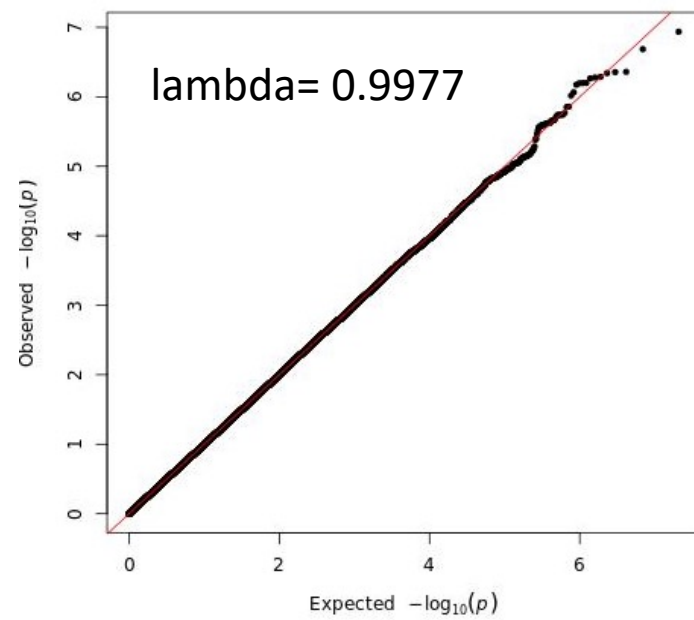

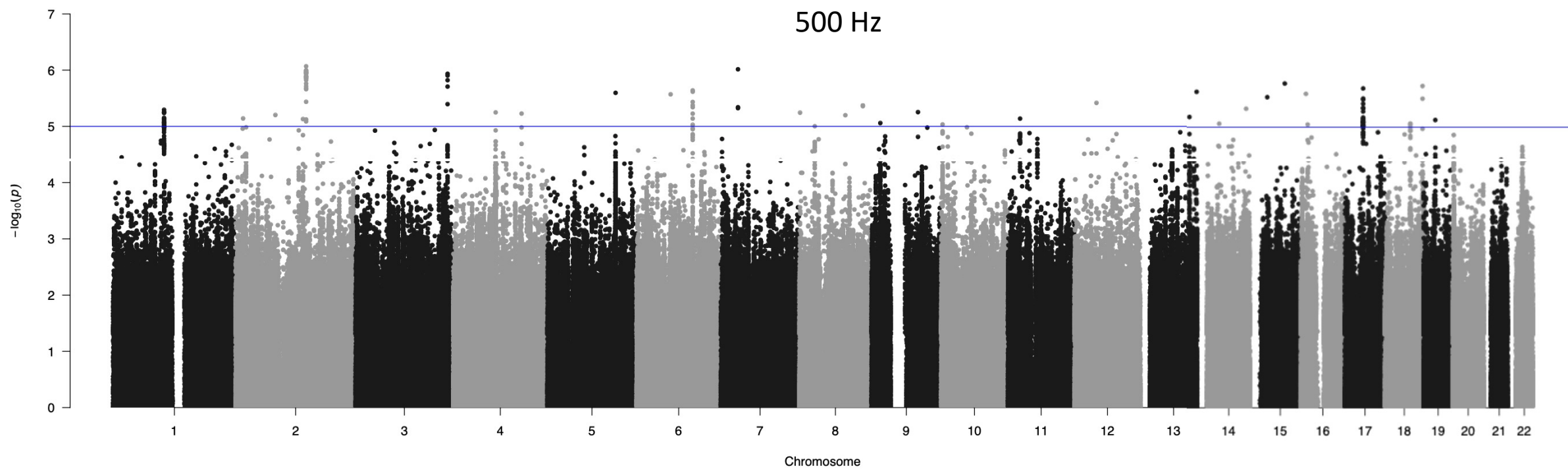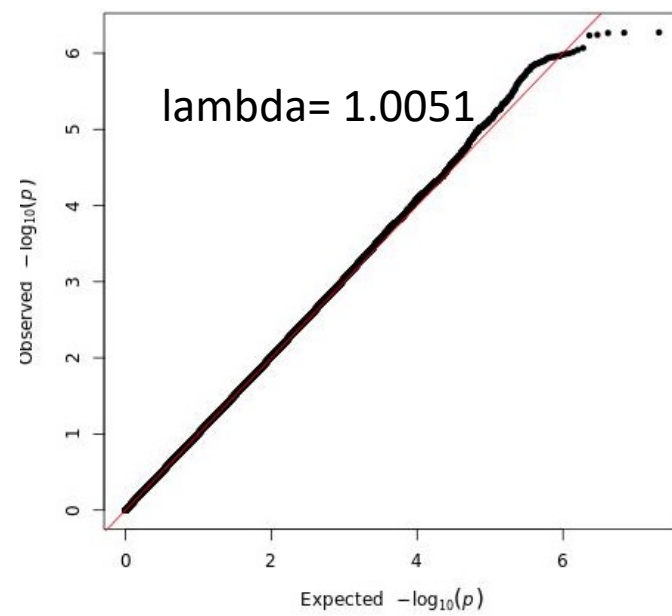

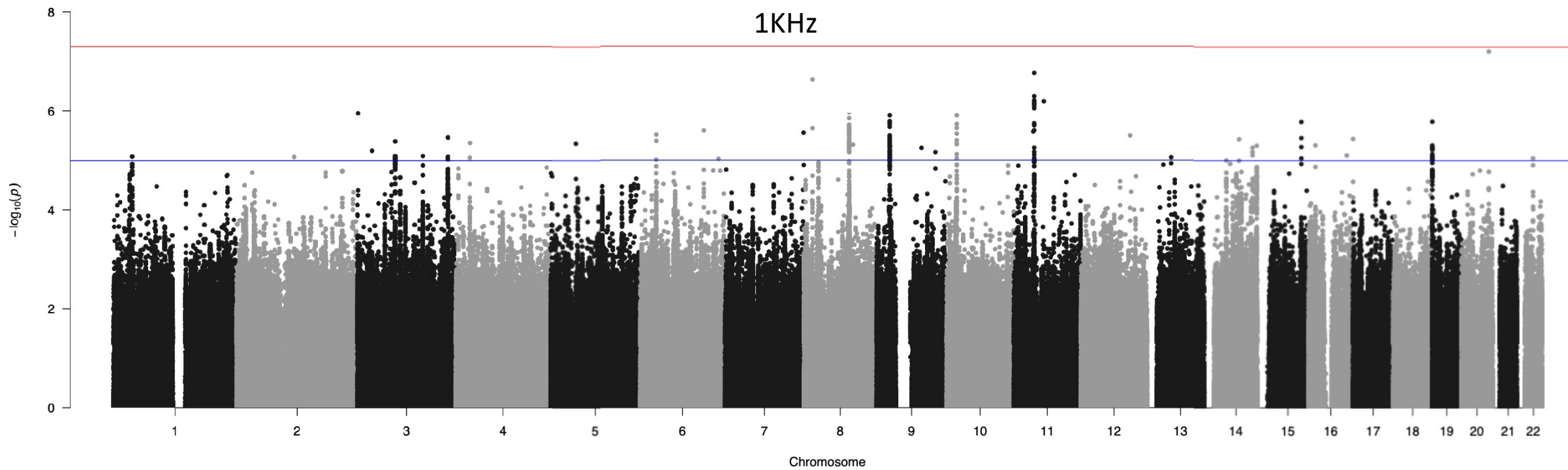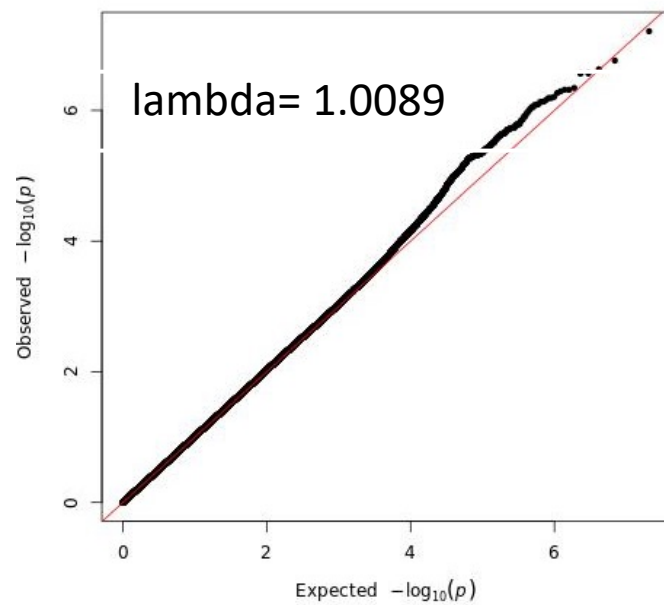

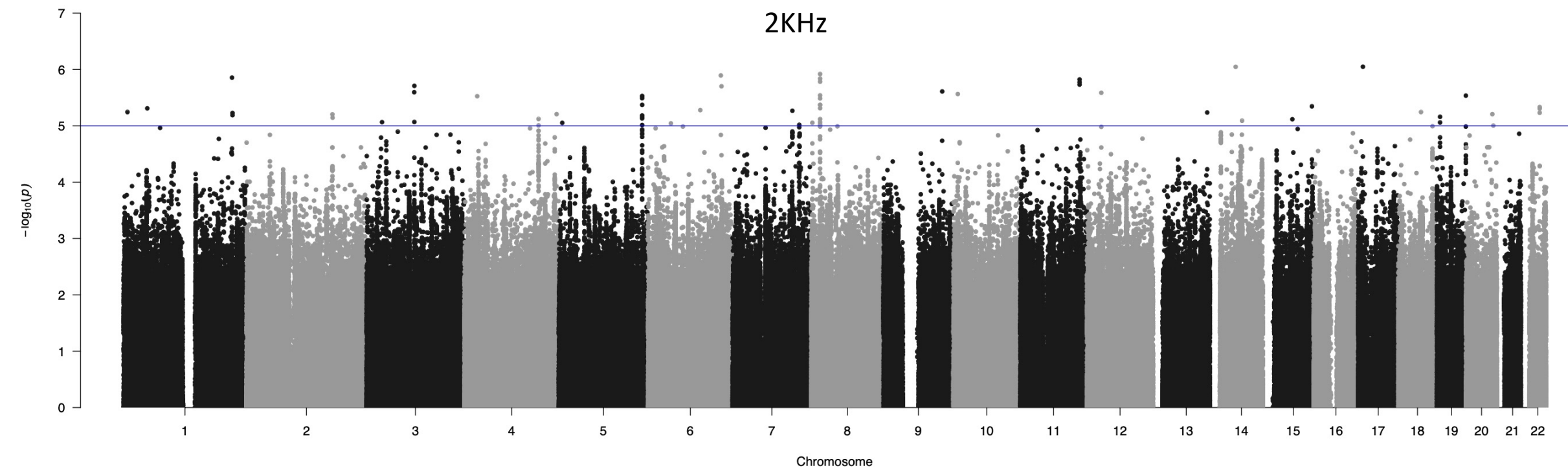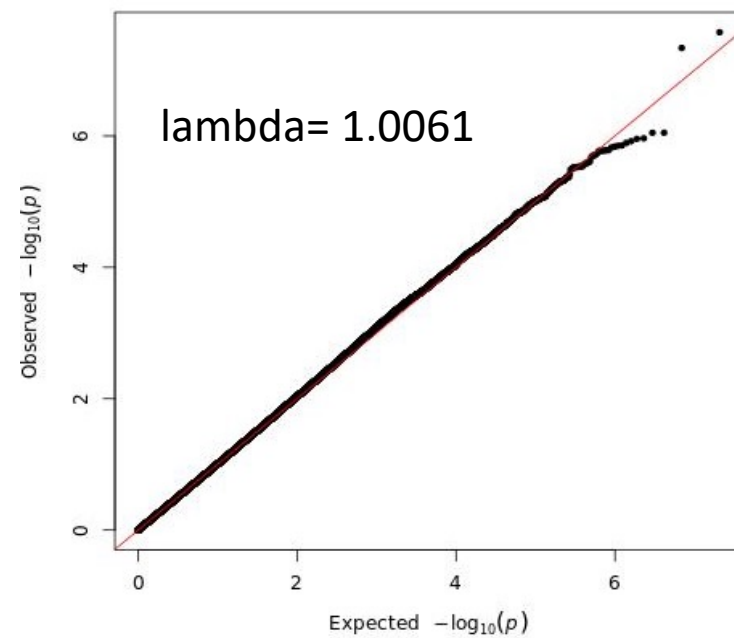

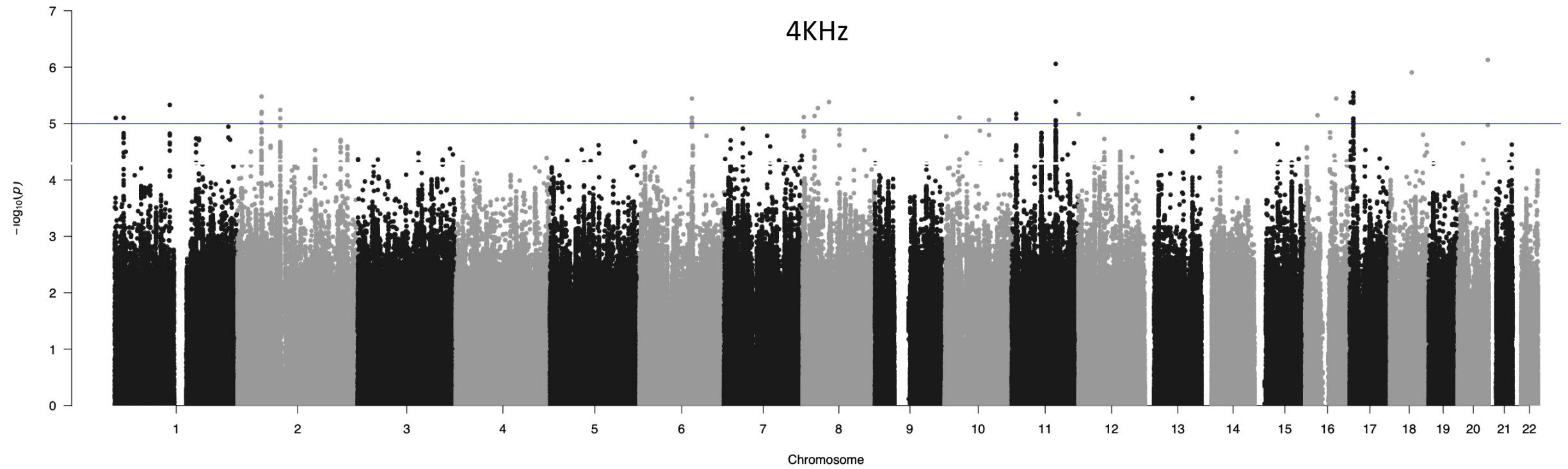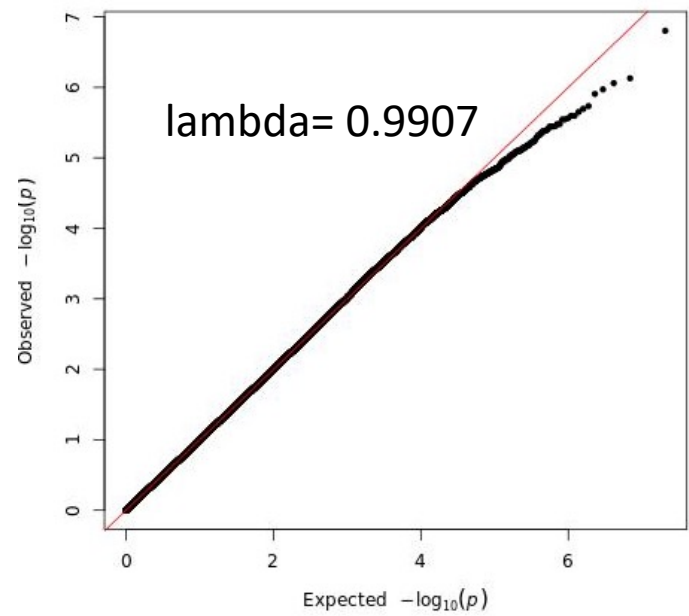

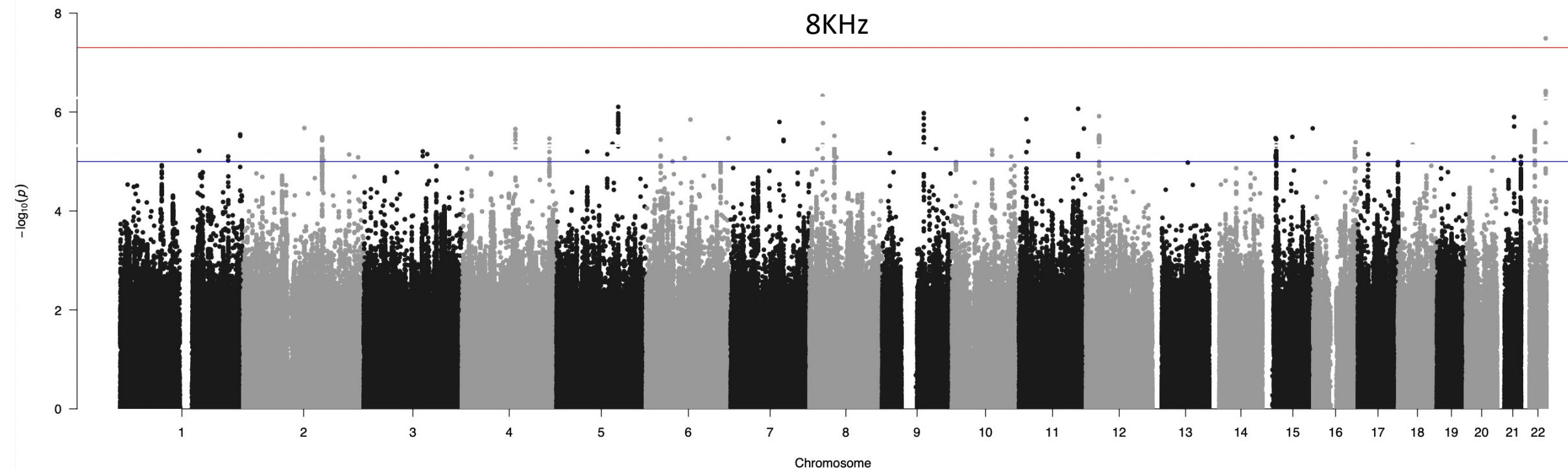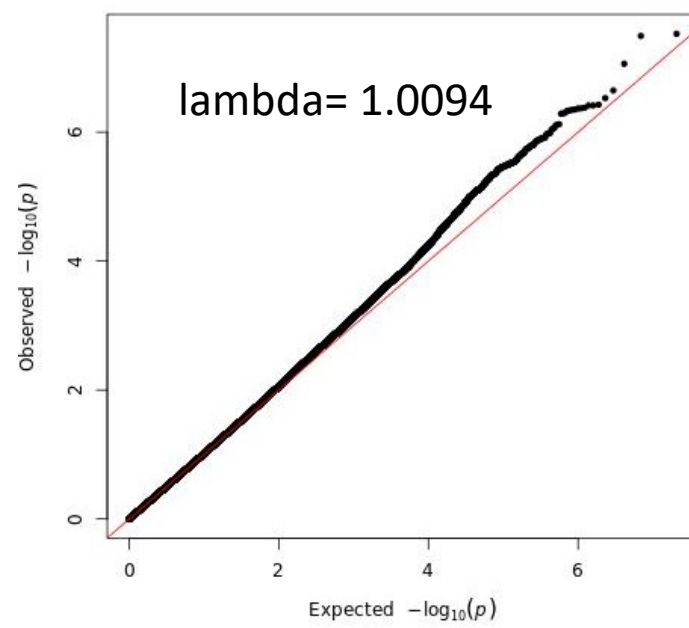

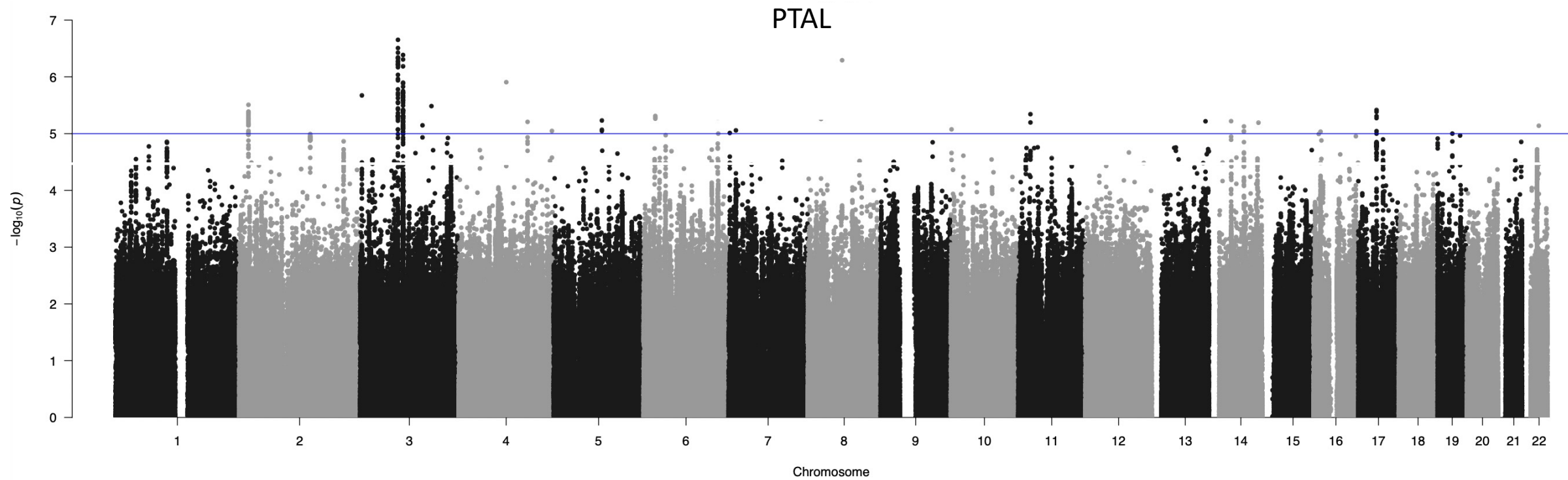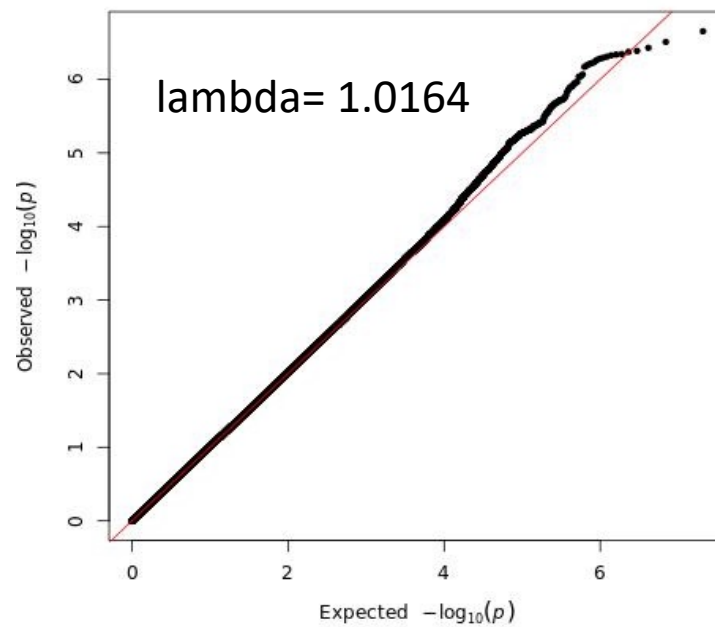

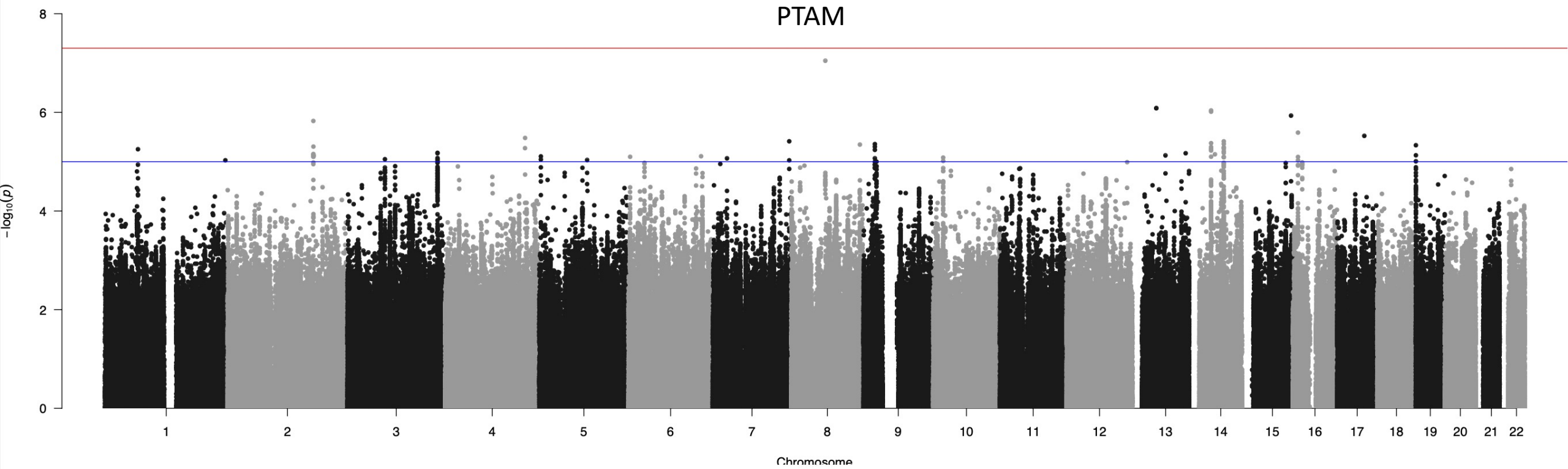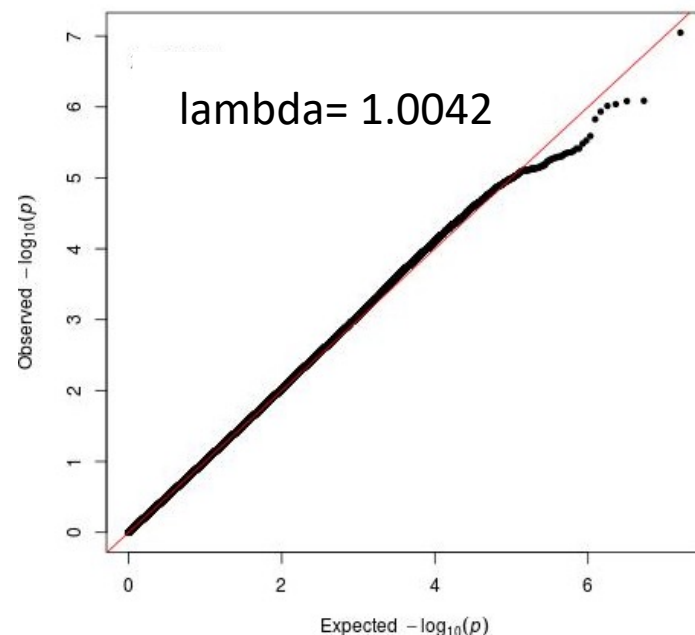

# PTAH

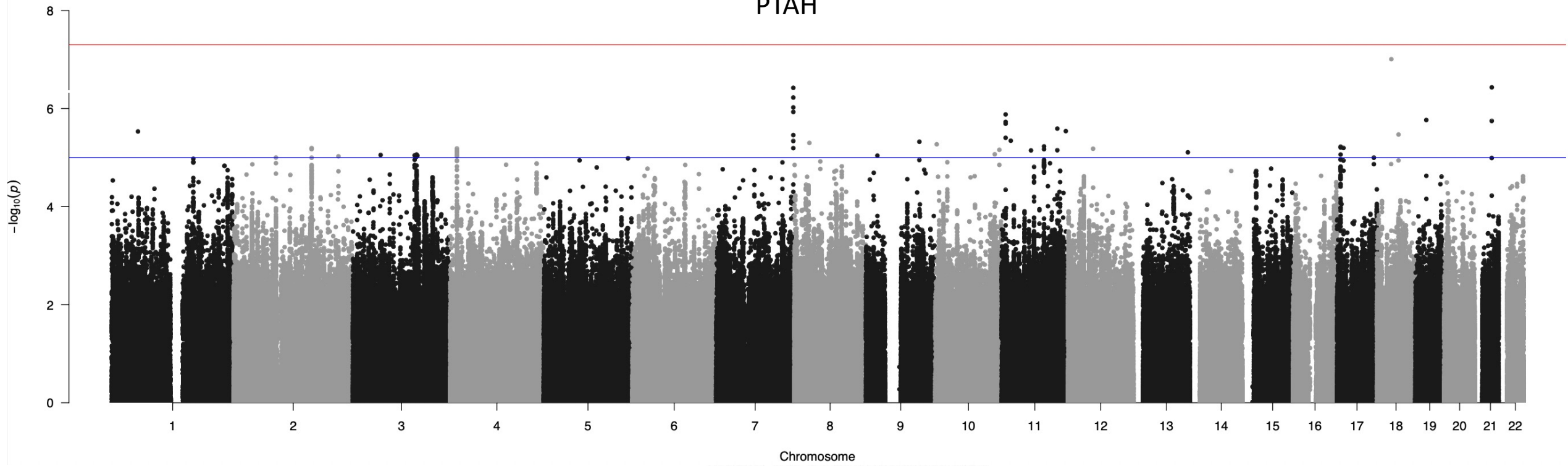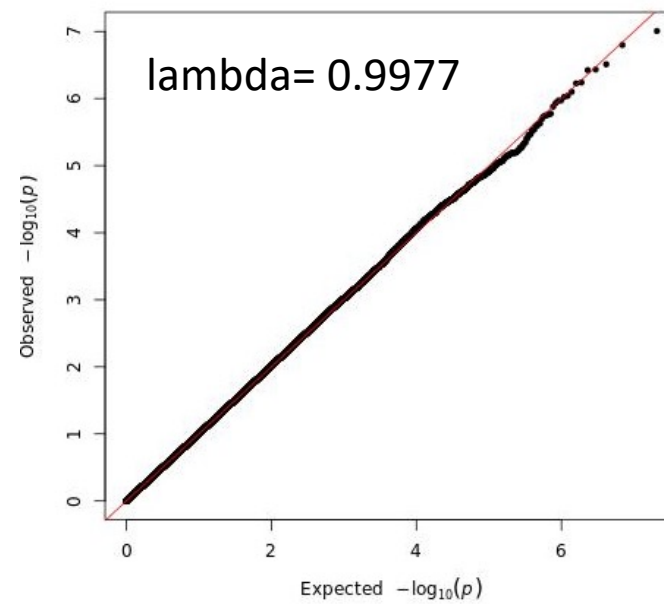

Supplement: Supplementary file 1 [file genes-12-01228-s001.zip › supplementary_files/Supplementary_figures.pdf]
